# Structural basis of the radical pair state in photolyases and cryptochromes†

**DOI:** 10.1039/d2cc00376g

**Published:** 2022-03-18

**Authors:** Andrea Cellini, Madan Kumar Shankar, Weixiao Yuan Wahlgren, Amke Nimmrich, Antonia Furrer, Daniel James, Maximilian Wranik, Sylvain Aumonier, Emma V. Beale, Florian Dworkowski, Jörg Standfuss, Tobias Weinert, Sebastian Westenhoff

**Affiliations:** Department of Chemistry and Molecular Biology, University of Gothenburg Box 462 40530 Gothenburg Sweden westenho@chem.gu.se; Division of Biology and Chemistry-Laboratory for Biomolecular Research, Paul Scherrer Institut 5232 Villigen Switzerland; Photon Science Division – Laboratory for Macromolecules and Bioimaging (LSB), Paul Scherrer Institut 5232 Villigen Switzerland; Photon Science Division – Laboratory for Synchrotron Radiation and Femtochemistry (LSF), Paul Scherrer Institut 5232 Villigen Switzerland; Department of Chemistry-BMC, University of Uppsala Husargatan 3 75237 Uppsala Sweden

## Abstract

We present the structure of a photoactivated animal (6-4) photolyase in its radical pair state, captured by serial crystallography. We observe how a conserved asparigine moves towards the semiquinone FAD chromophore and stabilizes it by hydrogen bonding. Several amino acids around the final tryptophan radical rearrange, opening it up to the solvent. The structure explains how the protein environment stabilizes the radical pair state, which is crucial for function of (6-4) photolyases and cryptochromes.

## Abstract 2

The structural response of the drosophila (6-4) photolyase to photoinduced electron transfer along a chain of tryptophans is revealed using a serial crystallographic snapshot of the protein in its radical pair state.

## Graphical abstract

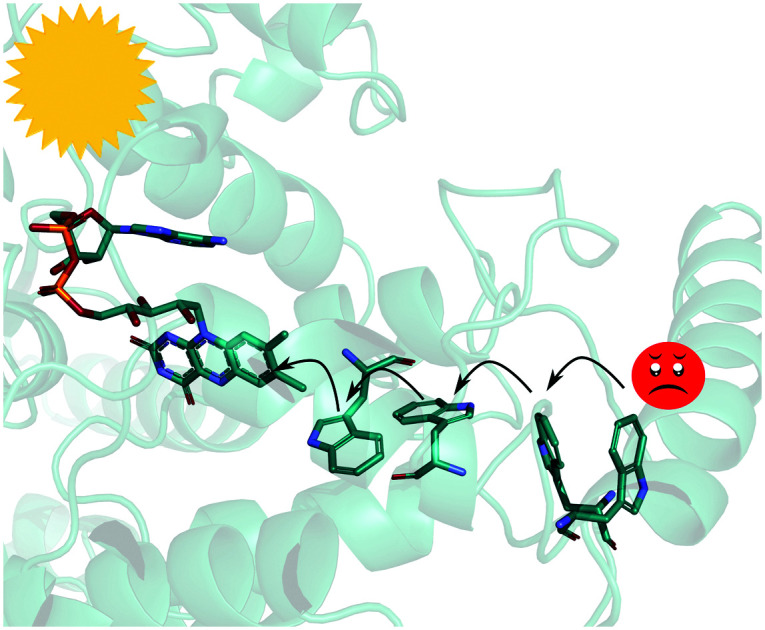

Photolyases are blue-light receptors, which repair damaged DNA using visible light.^1,2^ Two groups are distinguished according to their ability to repair Cyclobutyl Pyrimidine Dimers (CPD) lesions or (6-4) lesions. Photolyases are closely related to cryptochromes, which entrain the circadian clock^1^ and enable magnetoreception in some animals.^3^ Together, cryptochromes and photolyases form a superfamily, which is present in all kingdoms of life.^4^

Photolyases and cryptochromes share a Photolyase Homologous (PHR) domain, which holds a flavin adenine dinucleotide (FAD) as the main chromophore, a conserved triad/tetrad of tryptophans (Trp407, Trp384, Trp330, Trp381; all residue counts for the (6-4) photolyase from *Drosophila melanogaster*), and a binding site for the damaged DNA in photolyase (Fig. 1A).^5^

**Fig. 1:**
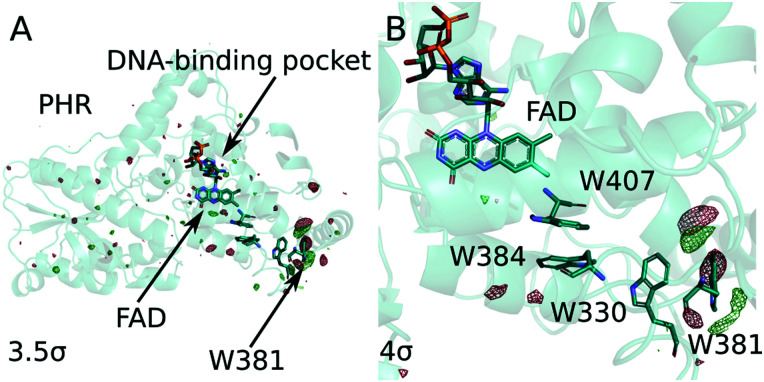
(A) Positive (green) and negative (red) electron density differences in *Dm*(6-4) photolyase are shown together with the protein structure in dark (pdb accession code: 7QUT). (B) The same data is shown for the tryptophan tetrad together with the FAD chromophore.

Signalling by cryptochromes and DNA repair by photolyases rely on photoinduced charge separation along the triad or tetrad of conserved tryptophans (Fig. 1B). The fully oxidized FAD is photoexcited and extracts an electron from the nearest tryptophan. The electron is then shuttled along the tryptophan triad/tetrad, yielding a radical pair consisting of semiquinone FAD˙^−^ and TrpH^+^.^6^ This state is remarkable, because the radicals are separated by more than 20 Å. In cryptochromes it is the active signalling state, providing for signalling in the circadian clock^7^ and possibly also for magnetovision.^8,9^ In photolyases it is the precursor state for a second photoreduction of FAD, producing doubly reduced FADH^−^, which in turn is necessary for DNA repair.^10^ The importance of the semiquinone radical pair is testified by that single point mutation on the last tryptophan into a redox-inactive phenylalanine hinders the DNA repair of photolyase, both *in vitro* and *in vivo*.^6,7,11^

It is expected that the protein has evolved to control the lifetime of the semiquinone radical pair state. In photolyases and cryptochromes the semiquinone radical pair is stabilized by deprotonation of the final TrpH˙^+^, yielding a neutral tryptophanyl radical (Trp˙). The FAD˙^−^:Trp381˙ state is stable for milliseconds to seconds.^12,13^ This FAD˙^−^:Trp381˙ state is thought to be important for sensing the magnetic field^14^ and the lifetime of this radical pair state appears to be controlled by partially conserved residues around the final tryptophan.^8^ In photolyases and some cryptochromes, the FAD˙^−^:Trp381˙ state further reacts by protonation of FAD˙^−^ to FADH˙. This process depends on the amino acid next to the N5 of the isoalloxazine ring of the FAD (Fig. 2A). An asparagine (Asn403) is conserved in photolyases and leads protonation on timescales of seconds.^15–17^ In plant cryptochromes an aspartate residue facilitates fast protonation within milliseconds,^18^ and in *Drosophila melanogaster* cryptochrome the closest residue to the main ring of the FAD is a cysteine, hindering the formation of FADH˙.^19,20^

**Fig. 2:**
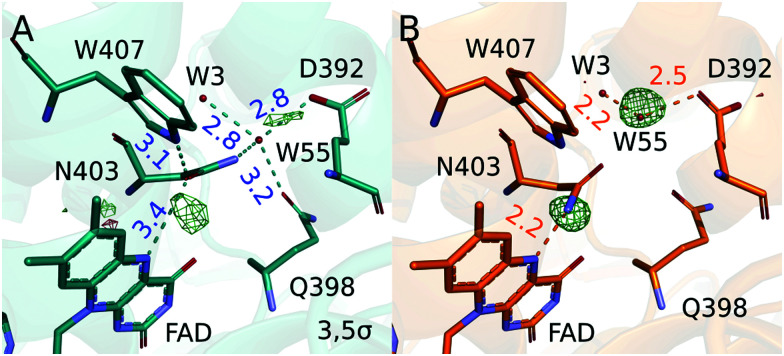
The observed (Δ*F*_o_) (A) and calculated (Δ*F*_c_) (B) difference electron densities around the FAD chromophore are shown. Distances between atoms are given in Angstrom.

The structures of photoylase and cryptochromes are solved in their resting states,^5,19–24^ but direct structural observation of the functional photoreduced states has not been achieved. Fourier transform infrared (FTIR) spectra suggest that structural changes occur in the protein when FAD is reduced and protonated to FADH˙, but the extend and specifics of these changes could not be determined.^25^ A combined spectroscopic and computational study of the *E.coli* CPD photolyase in the same state suggests that a hydrogen bond is formed between the carbonyl group of the Asn403 side chain and the N5 of FAD, thereby stabilizing the protonated FADH˙.^26^ The isoalloxazine ring of the flavin chromophore is expected to bend when photoreduced.^27^ Overall, these changes are probably rather small,^26–28^ especially when compared to chromophores in other photosynthetic or signalling proteins, such as rhodopsins,^29^ phytochrome^30^ or photoactive yellow protein.^31^ Despite these studies the structural mechanism with which the protein guides the function of cryptochromes and photolyase remains unclear. More specifically, it is not clear how the proteins facilitate DNA repair of (6-4) lesions, whether the proton from TrpH˙^+^ is donated to an internal proton acceptor or to the solvent, and how the protein rearranges to stabilize the long-range radical pair.

Here, we report a serial X-ray crystallographic snapshot of the photoexcited (6-4) photolyase from *Drosophila melanogaster* (*Dm*(6-4)photolyase) in the semiquinone radical pair state. We employed synchrotron-based serial millisecond crystallography at beamline X06SA at the Swiss Light Source, with microcrystals of *Dm*(6-4) photolyase supplied to the X-ray beam in hydroxyethylcellulose using a high viscosity extruder.^32,33^ The photolyase is photoactive in the microcystals as reported earlier.^34^ The sample was excited with a fibre-coupled CW solid state laser at 473 nm, while extruding from the nozzle to the interaction point with the x-ray beam (X-ray spot size was 5 × 15 μm^2^). The speed of the viscous jet was 0.001 μl s^−1^, which results in a travel time in the x-ray beam of approximately 0.3 second. 64 785 and 82 895 diffraction patterns were indexed for the unexcited (dark) and photoexcited (light) sample, respectively. The patterns were indexed, scaled and merged using CrystFEL.^35^ The difference electron density maps were computed using the script fobs_minus_fobs in PHENIX.^36^

The real-space map of the resulting difference structure factor (Δ*F*_o_ = *F*_o_(light) − *F*_o_(dark)) is presented Fig. 1. It shows prominent features at and around Trp381 (Fig. 1B). The signals in the chromophore region are weaker and sparser, but above the noise (Fig. 2A). We do not observe significant signals around the other tryptophans of the tetrad (Fig. 1B). The changes around the fourth tryptophan were reproduced in two other data sets recorded with illumination at 488 nm and 455 nm, albeit with weaker signal intensity (data not shown). Consistent with the illumination of the protein for approximately 0.3 second,^12^ we assign the observed changes to the radical pair state FAD˙^−^:Trp381˙, which includes the deprotonated tryptophan.

We modelled the structural changes using real space refinement in coot against 2*F*_e_ − *F*_c_ maps computed from extrapolated structure factors (*F*_e_ = *F*_o_(dark) + 1/*r*Δ*F*_o_).^29,31^ A photoactivation of *r* = 14% was estimated on the assumption that the electron densities in the extrapolated map should not be negative (see ESI† for analysis). The validity of the structural model of the light state was confirmed by comparing calculated to observed difference electron density maps (Fig. 2 and 3). The calculated difference map was computed from the Fc's of the model in dark and light as (Δ*F*_c_ = *F*_c_(light) − *F*_c_(dark)).

**Fig. 3:**
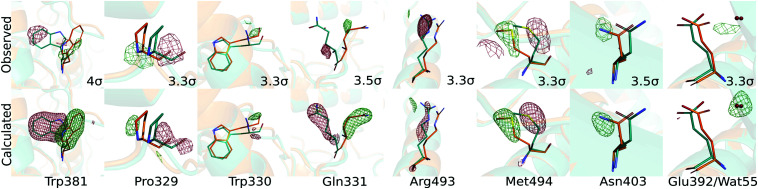
Comparison of the observed (Δ*F*_o_) (upper row) and the calculated (Δ*F*_c_) (lower row) difference electron densities verifies the structural model of the semi-reduced photolyase.

In the chromophore region (Fig. 2A), two positive peaks between the N5 of FAD and conserved Asn403 and in between water 55 and Glu392 are the most prominent difference electron density features. The corresponding negative density on the Asn403 side chain is weaker and broader and thus not seen at the contour level in Fig. 2A. We also do not observe clear signatures of bending of the chromophore, contradicting expectations,^27^ in agreement with a recent crystallographic study of a photodecarboxylase.^37^ This may be due to disorder in the dark state, overlap of atomic positions in the dark and light state, or compensating effects of waters entering the space. In the dark, Asn403 has hydrogen bonds to water 55, Gln398 and to Trp407, which is the first tryptophan in the tetrad. After illumination and reduction of FAD the side chain of Asn403 moves into close hydrogen bonding distance (2.2 Å) to the N5 of FAD˙^−^. This is indicated by the positive difference electron density between Asn403 and FAD (Fig. 2B). We propose that the side chain of Asn403 flips in this process. The electron densities maps are not sensitive to the flip at the obtained resolution, but the nature of hydrogen bonds that the asparagine can form call for this suggestion. In the dark, the amide oxygen faces the Trp407 as a hydrogen bond acceptor, while the amide nitrogen faces the N5 of FAD to donate a hydrogen bond to the photoreduced FAD. In this process, the hydrogen bonds to Trp407 and water 55 are broken. The water moves by 1.1 Å away from its position in the dark, loosing connection Gln398 and binding tighter to its remaining binding partners Glu392 and water 3 (Fig. 2B). This movement is supported by the observed positive difference electron density between water 55 and Glu392. The data establishes that Asn403 actively stabilizes the negative charge on FAD˙^−^ by donating a hydrogen bond to its N5.

The observations underpin the central role that Asn403 plays in photolyases. While the asparagine is not as effective in donating a proton to the reduced FAD as the corresponding aspartate in plant cryptochromes, it is able to stabilize the reduced FAD through hydrogen bonding. This has also been concluded from recent computational study, where a conformation similar to our observed structure was assigned the lowest free energy.^15^ Considering that the hydrogen bond between Asn403 and the first tryptophan in the tetrade (Trp407) is broken, it is tempting to assume that the Asn403 side chain already moves in the first step of the electron transfer chains. However, this question require further investigation.

Next, we focus on the region around Trp381. The comparison of the dark and the light structure shows movements of Trp381, Pro329, Arg493, Met494, and Gln331 (Fig. 4A). The residues are at least partially conserved among photolyases (Fig. S2, ESI†), indicating that they are evolutionary important for their function. In the dark structure, the carboxyl group and indole functional group of Trp381 are within hydrogen bond distance to the amino group of Gln331 and the backbone C = O of Pro329, respectively (Fig. 4). In the light structure the indole group of Trp381 rotates about 34° towards the solvent. The solvent-accessible surface for Trp381 increases from 2.1 Å in dark to 51 Å in light, as estimated by AREAIMOL (ccp4i). The movement leads to an increase of the distance to the FAD chromophore (measured between N10 of FAD and the indole N of Trp) from 23 Å in dark to 24 Å in light. This change may slow down charge recombination. The hydrogen bond to Pro329 is broken and the proline moves 0.7 Å away from its previous position (Fig. 4A). Trp381 also looses its hydrogen bond to the amino group of Gln331, and the side chain of Gln331 swings out to into the solvent area. This creates space for the movement of Trp381 (Fig. 4). Together with the observed changes of Arg493, the surrounding residues contribute to accommodate the positional change of the indole group of Trp381, which is further exposed to the solvent after illumination (Fig. 4B).

**Fig. 4:**
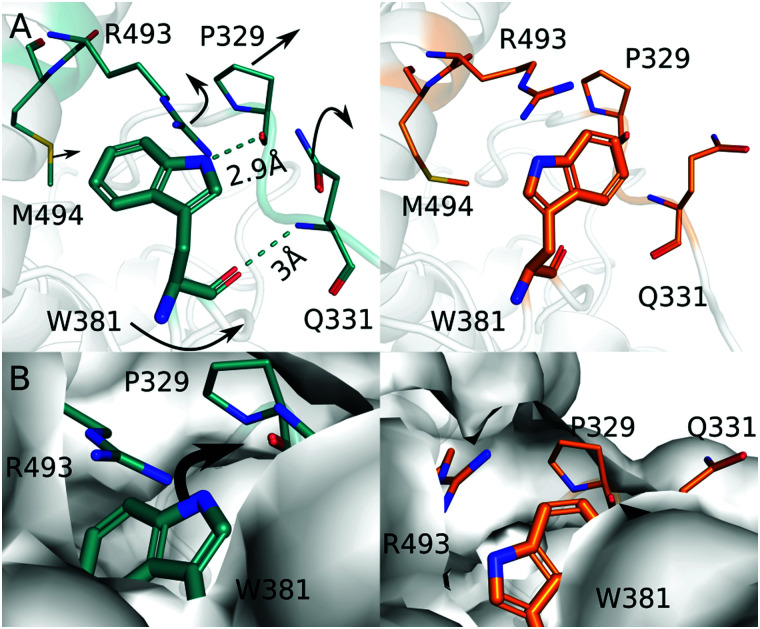
(A) The dark (blue) and the light (orange) structure around Trp381 are depicted to highlight the difference in structure before and after light exposure. (B) Surface representations of the dark and the light structure are shown. Moving from the dark structure (blue) to the light structure (orange), the solvent accessible around Trp381 is increased.

Based on these observations, we propose that the photooxidation of Trp381 first leads to breaking of the hydrogen bond with Pro329. Pro329 and Gln331 are located on the same loop, which is now more flexible to move and therefore Gln331 also disconnects from Trp381. This is a prerequisite for the indole group of Trp381 to gain solvent access for deprotonation (Fig. 4B). We consider that the movements of Arg493 and Met494, which are part of the so-called 22 α-helix, occur in response to this action. Based on hydrogen exchange mass spectrometry in *Chlamydomonas reinhardtii* cryptochrome, it was proposed that these structural elements get disrupted or move when the final tryptophan is deprotonated.^38^ Our structural observation confirms this, even though only small movements of the 22 α-helix are observed here.

An important question is whether it is a residue or the solvent that takes up the proton from Trp381H˙^+^. We do not observe a residue or localized water molecules nearby the final tryptophan that might act as proton acceptor and we find that the tryptophan swings outwards towards the solvent (Fig. 4B). Therefore, we conclude that it is the solvent that extracts the proton from the final and fourth tryptophan. This is different to proposals for photolyases with tryptophan triade. In the *Arabidopsis Thaliana* (6-4) photolyase, a cysteine relays the proton to the solvent^39^ and in a class II CPD photolyase, a conserved tyrosine and a network of bound water molecules facilitate deprotonation.^40^ The third tryptophan is typically buried in the protein, and therefore residues that relay the proton are needed.

We present the structure of a (6-4) photolyase in the photoactivated FAD˙^−^:Trp˙ state. The observed radical pair state is the precursor state for DNA repair and it is similar to the signalling state in cryptochromes. The changes in the vicinity of the FAD and Trp381 both act to stabilize the radical pair that forms on these two residues. Our crystals do not contain DNA and as such we do not observe DNA repair directly. Nevertheless, we find how the protein prepares for this function. Compared to other photoactive proteins, we observe rather small structural changes around the chromophore, with Asn403 establishing a tight hydrogen bond to the N5 of the FAD as most prominent feature. This is consistent with a small reorganization energy of the chromophore, yet the protein achieves significant stabilization of the radical. We consider that this is beneficial for the function of photolyases, because the protein needs to make use of as much of the photon energy as possible for efficient DNA repair. It is possible to co-crystallize photolyases and DNA^23,24^ and in future studies DNA repair could be monitored using approaches that build on this study.

S. W. conceived the project, A. C. produced the crystals, all authors performed the experiment, A. C., A. N., M. K. S., W. Y. W., T. W. and S. W. analysed the data, A. C. and S. W. wrote the paper with input from all authors.

## Conflicts of interest

There are no conflicts to declare.

## Supplementary Material

CC-058-D2CC00376G-s001
