# Supplementary material for: Structural basis of the radical pair state in photolyases and cryptochromes
Source: Chem Commun (Camb). 2022 Mar 18;58(31):4889–92. doi: 10.1039/d2cc00376g (PMC9008703; doi:10.1039/d2cc00376g)
Supplement: CC-058-D2CC00376G-s001 [file CC-058-D2CC00376G-s001.pdf]

Electronic Supplementary Material (ESI) for ChemComm.
This journal is © The Royal Society of Chemistry 2022

Electronic Supplementary Information

Structural basis of the radical pair state in photolyases and cryptochromes.

Andrea Cellini,[a] Madan Kumar Shankar,[a] Weixiao Yuan Wahlgren[a], Amke Nimmrich[a], Antonia Furrer[b], Daniel James[b], Maximilian Wranik[b], Sylvain Aumonier[c], Emma V. Beale[d], Florian Dworkowski[c], Jörg Standfuss[b], Tobias Weinert[b] and Sebastian Westenhoff,[*a,e]

[a] *Department of Chemistry and Molecular Biology, University of Gothenburg Box 462, 40530 Gothenburg, Sweden*

[b] *Division of Biology and Chemistry-Laboratory for Biomolecular Research, Paul Scherrer Institut, 5232 Villigen, Switzerland*

[c] *Photon Science Division - Laboratory for Macromolecules and Bioimaging (LSB), Paul Scherrer Institut, 5232 Villigen, Switzerland*

[d] *Photon Science Division - Laboratory for Synchrotron Radiation and Femtochemistry (LSF), Paul Scherrer Institut, 5232 Villigen, Switzerland*

[e] *Department of Chemistry-BMC, University of Uppsala, Husargatan 3, 752 37 Uppsala*

[*] To whom correspondence should be addressed; E-mail: westenho@cmb.gu.se

Experimental details

Protein expression and crystallyzation

Dm(6-4) photolyase gene was codon optimized and ordered at GenScript. The gene was inserted between NcoI and XhoI in pET21d (+) plasmid with a stop-codon before the C-terminal His-tag sequence. The plasmid was transformed in BL21(DE3) cells and grew in a Studier medium[1]with additional 50 μg/mL carbenicillin for 2-3 hours at 37 ° and then overnight at 20 °. Cells were lysated with sonication (Q700 sonicator, Qsonica) with the settings: 30 % amplitude, pulse-on time 10 second, pulse-off time 30 second and process time of 10 min. The protein was purified through Hi-trap Heparin Column purification (Ge Healthcare) followed by size exclusion chromatography (Hiload Superdex 16/600 200 pg, Ge Healthcare)[2]. The protein concentration was estimated by determining the FAD concentration and the photoconversion of the protein was evaluated by illuminating the sample with a 445 nm LED[3]. All the experimental procedures were performed under safe red light.

Batch crystallization

The crystals were first grown at 4 ° C in hanging drop plates with a reservoir of 100 mM bis-tris pH=6.5, 200 mM lithium sulphate monohydrate, 22 % PEG 3350, 0.5 % Ethyl acetate. After two days, the macrocrystals were crushed and used as seeds for batch crystallization[3] . Prior to collecting data, the crystals were spun down, 90 % of the mother liquor was removed and the content of the vial was mixed in 1:3 ratio with hydroxy ethyl cellulose (HEC) matrix. HEC matrix was produced by dissolving 22 % of HEC in water (w/w).

Data collection

The data were collected at beamline X06SA at the Swiss Light Source Synchrotron (SLS). The sample was extruded through a high viscosity injector with a 75 μm diameter nozzle[4]. The sample flow rate was set to 0.08 μl/min (300 μm/s) for both dark and light steady state data sets. For the light data set, a CW 473 nm laser(Crystalaser CL-473-150) with a fluence of 612 mW/mm2 was employed. From the flow speed and distance between nozzle tip and x-ray interaction point (90 μm) we estimate that the sample was illuminated for approximately 0.3s before being probed by the x-ray. The sample was prepared and data were collected at room temperature.

Data analysis

The diffraction images were indexed, integrated and merged with Crystfel 0.9.1[5]. Indexamajig was used for indexing patterns with xgandalf[6]. These patterns had indexing ambiguity issues related to the tetragonal Bravais lattice of the crystals. This issue was addressed and corrected with ambigator by using the operator h,k,-l. The resulting stream file was merged, scaled and post-refined with partialator. The partial reflection were treated using the unity model in partialator. The dark state structure is phased using MR (PDB ID: 7AYV), The structure was further refined using Phenix[7] and subsequently modelled with COOT[8].

Difference maps (Fobsdark-Fobslight) were computed using PHENIX's Isomorphous difference map module[7] and loaded in Coot[8] as reference for residues position refinement for the light structure. The extrapolated structure factors were calculated as follows: $(Fe = Fo(dark) + 1/r\Delta Fo$[9,10].

The extrapolated maps were used to estimate the percentage of activation (the percentage of the protein that get excited by the laser). The mean electron density of the residues that we see changes occurring at and the mean negative features at 3 rmsd were plotted against different level of activa-

tion (Fig.S1)[11]. The point at which the two plots intersects gives us the percentage of protein that is activated by light. The activation factor was deemed to be 14 %. The extrapolated map was used to refine the light activated structure in COOT[8]. Further, the calculated difference maps were plotted using the reported methods[12].

The dark structure has been deposited in PDB with the 7QUT ID, whereas the light structure can be found in the CXIDB (ID 202) .

UV/vis spectroscopy UV/VIS absorption measurements of Dm(6-4)photolyase in solution were performed to investigate which state is reached by illumination. The sample was excited for 100 ms with light from an LED at 453 nm and spectra were recorded 5 ms after the end of the illumination. The results are presented in Fig. 3, panel A shows the raw spectra for illuminated and dark measurement. Panel B shows the background corrected spectra, calculated from the spectra in panel A. This corresponds to the absorption spectra of Dm(6-4)photolyase before and after illumination. For the dark measurement (blue) we observe a good agreement with the expected absorption profile for a photolyase in the oxidized FAD state and a reduced state in light (red). Panel C shows absorption spectra of the different oxidation states of the FAD taken from literature.[13] Panel D displays the difference absorption spectrum between light and dark (blue) which was computed by subtracting the two spectra in panel A. A matrix division was performed using the spectra in C and this yielded the contribution of the states to the difference scattering (see inset). The resulting difference spectrum is shown in red. This verifies that after illumination for 100ms the photolyase is in the semi-reduced $FAD^{\cdot -}$ state. The minor negative contribution of FADH is attributed to uncertainties in the measurement.

Table 1 Data and refinement statistics.

	dark	light
PDB code	7QUT	
Space group	P 41	P41
Cell constants		
a, b, c (Å)	103.60 103.60 52.08	103.60 103.60 52.08
α, β, γ (°)	90.0 90.0 90.0	90.0 90.0 90.0
Resolution (Å)†	19.30-2.24 (2.24-2.26)	19.30-2.50 (2.50-2.52)
Data completeness (%)†	100 (100)	100 (100)
R_{split}(%) $^{\dagger *}$	9.53 (151.63)	7.87 (72.15)
CC* $^{\dagger 1}$	0.99(0.58)	0.99 (0.34)
CC1/2 [2]	0.96 (0.20)	0.96 (0.06)
$< I/\sigma(I) >$ †	8.58 (0.71)	13.95 (1.41)
Multiplicity †	1948	2902
Number of hits	461638	829692
Number of indexed hits	64785	82895
Number of total reflection	52396029	56202479
Number of unique reflections	26853	19365
Refinement		
R_{work}/R_{free}	0.15/0.19	0.41/0.44 [3]
Wilson B-factor (Å^2)	41.6	n/a
Total number of atoms	4277	4277
Average B, all atoms (Å^2)	59.72	60.05
R.m.s deviations		
Bond lengths (Å)	0.007	0.014
Bond angle (°)	1.56	1.53

† Highest resolution shell is shown in parenthesis.

$*$ Rsplit= $1/\sqrt{2\frac{\sum hkl|Ieven-Iodd|}{1/2\sum hkl|Ieven+Iodd|}}$

1 CC*=$\sqrt{\frac{2CC1/2}{1+CC1/2}}$

2 CC1/2= $\frac{\sigma t^2}{\sigma t^2+\sigma e^2}$

where σt^2 is the variance of the difference between the intensities and their average and σe^2 is the variance of random error of merged half datasets.

3 The R values are not meaningful because the structure was refined only in real space and only in selected areas.

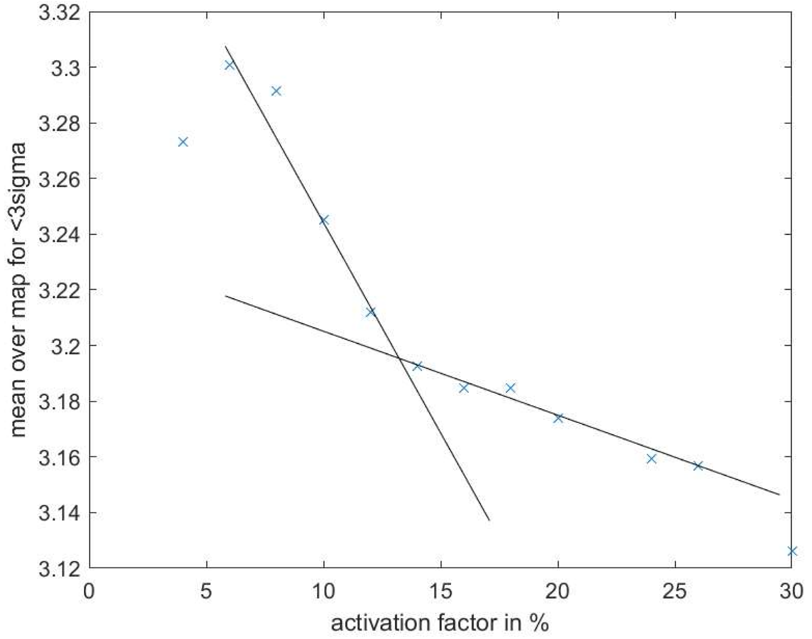

Fig.S 1 Negative features plotted in function of the percentage of activation. The point of intersection determines the level of activation. In our case, the activation level is about 14%

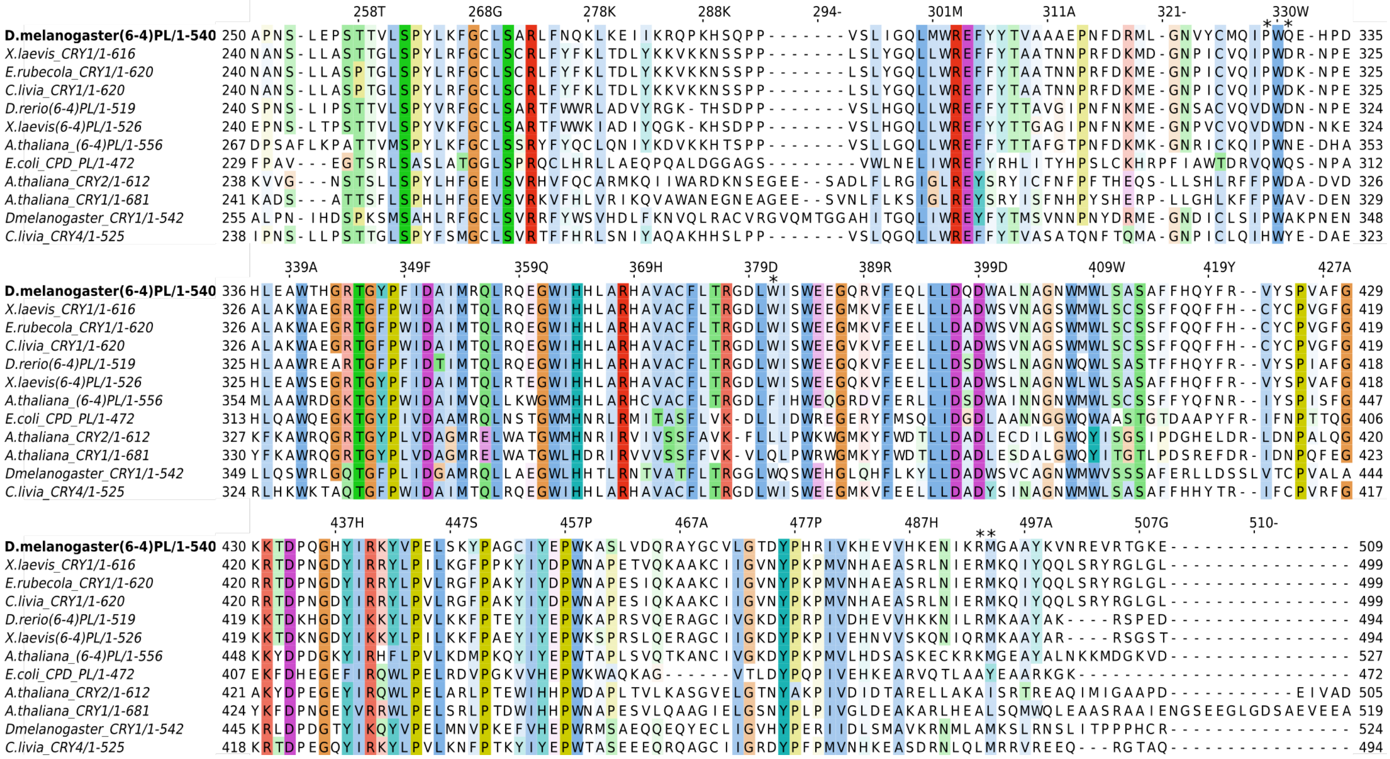

Fig.S 2 Alignment of the C-terminal region of photolyases and cryptocrome. The alignment was performed in Jalview 2.11.1.4 with Clustal omega[14] . Residues conserved more than 25% are coloured according to clustal color scheme.The asterisks are placed on top of residues which show difference signal in our data.

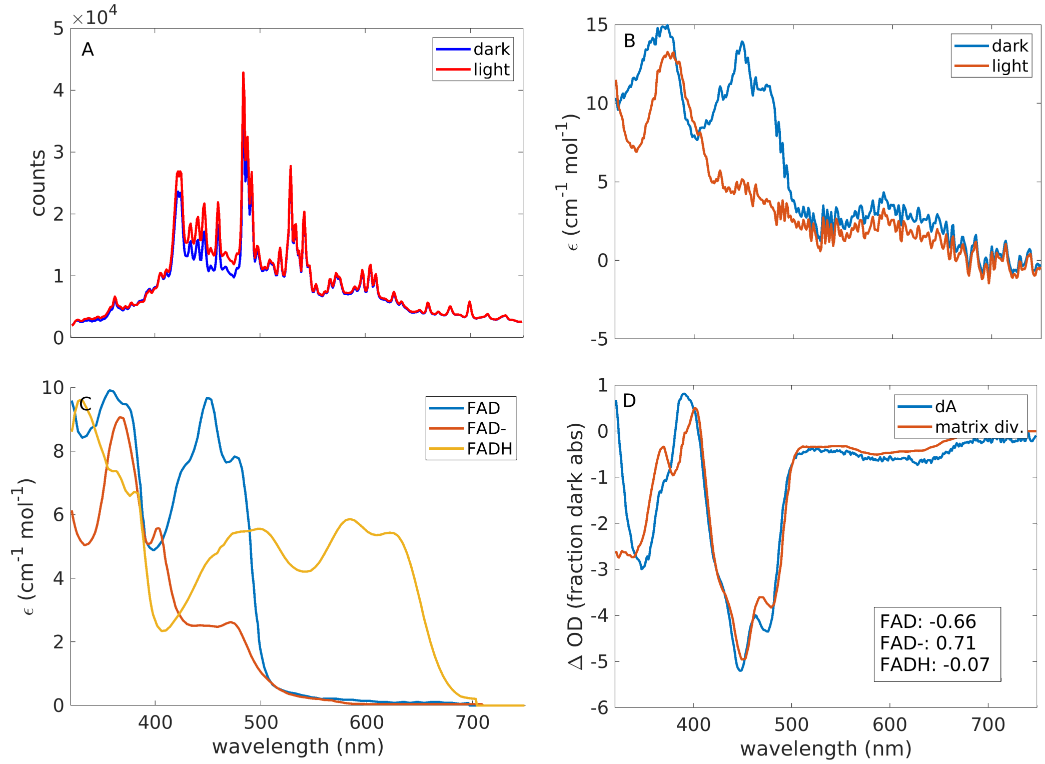

Fig.S 3 Results from the UV/Vis spectroscopy measurements. A: Raw spectra, B: absorption spectra before and after illumination, C: absorption spectra of different oxidation states of FAD from [13], D: difference spectrum from experiment (blue) and result from matrix division (red). For details see text.

Notes and references

1 F. W. Studier, *Protein expression and purification*, 2005, **41**, 207–234.

2 M. J. Maul, T. R. Barends, A. F. Glas, M. J. Cryle, T. Domratcheva, S. Schneider, I. Schlichting and T. Carell, *Angewandte Chemie International Edition*, 2008, **47**, 10076–10080.

3 A. Cellini, W. Yuan Wahlgren, L. Henry, S. Pandey, S. Ghosh, L. Castillon, E. Claesson, H. Takala, J. Kübel, A. Nimmrich *et al.*, *Acta Crystallographica Section D: Structural Biology*, 2021, **77**, 1001–1009.

4 U. Weierstall, D. James, C. Wang, T. A. White, D. Wang, W. Liu, J. C. Spence, R. B. Doak, G. Nelson, P. Fromme, R. Fromme, I. Grotjohann, C. Kupitz, N. A. Zatsepin, H. Liu, S. Basu, D. Wacker, G. Won Han, V. Katritch, M. Boutet, Sébastien Messerschmidt, G. J. Williams, J. E. Koglin, M. M. Seibert, M. Klinker, C. Gati, R. L. Shoeman, A. Barty, H. N. Chapman, R. A. Kirian, K. R. Beyerlein, D. Stevens, Raymond C. Li, S. T. A. Shah, N. Howe, M. Caffrey and V. Cherezov, *Nature communications*, 2014, **5**, 1–6.

5 T. A. White, R. A. Kirian, A. V. Martin, A. Aquila, K. Nass, A. Barty and H. N. Chapman, *Journal of applied crystallography*, 2012, **45**, 335–341.

6 Y. Gevorkov, O. Yefanov, A. Barty, T. A. White, V. Mariani, W. Brehm, A. Tolstikova, R.-R. Grigat and H. N. Chapman, *Acta Crystallographica Section A: Foundations and Advances*, 2019, **75**, 694–704.

7 D. Liebschner, P. V. Afonine, M. L. Baker, G. Bunkóczi, V. B. Chen, T. I. Croll, B. Hintze, L.-W. Hung, S. Jain, A. J. McCoy *et al.*, *Acta Crystallographica Section D: Structural Biology*, 2019, **75**, 861–877.

8 P. Emsley, B. Lohkamp, W. G. Scott and K. Cowtan, *Acta Crystallographica Section D: Biological Crystallography*, 2010, **66**, 486–501.

9 P. Nogly, T. Weinert, D. James, S. Carbajo, D. Ozerov, A. Furrer, D. Gashi, V. Borin, P. Skopintsev, K. Jaeger, K. Nass, P. Båth, R. Bosman, J. Koglin, M. Seaberg, T. Lane, D. Kekilli, S. Brünle, T. Tanaka, W. Wu, C. Milne, T. White, A. Barty, U. Weierstall, V. Panneels, E. Nango, S. Iwata, M. Hunter, I. Schapiro, G. Schertler, R. Neutze and S. Jörg, *Science*, 2018, **361**, eaat0094.

10 K. Pande, C. D. Hutchison, G. Groenhof, A. Aquila, J. S. Robinson, J. Tenboer, S. Basu, S. Boutet, D. P. DePonte, M. Liang *et al.*, *Science*, 2016, **352**, 725–729.

11 S. Pandey, R. Bean, T. Sato, I. Poudyal, J. Bielecki, J. C. Villarreal, O. Yefanov, V. Mariani, T. A. White, C. Kupitz *et al.*, *Nature methods*, 2020, **17**, 73–78.

12 E. Claesson, W. Y. Wahlgren, H. Takala, S. Pandey, L. Castillon, V. Kuznetsova, L. Henry, M. Panman, M. Carrillo, J. Kübel, R. Nanekar, L. Isaksson, A. Nimmrich, A. Cellini, D. Morozov, M. Maj, M. Kurttila, R. Bosman, E. Nango, R. Tanaka, T. Tanaka, L. Fangjia, S. Iwata, S. Owada, K. Moffat, G. Groenhof, E. A. Stojković, S. M. Ihalainen, Janne A. and S. Westenhoff, *Elife*, 2020, **9**, e53514.

13 P. Müller, E. Ignatz, S. Kiontke, K. Brettel and L. O. Essen, *Chemical Science*, 2018, **9**, 1200–1212.

14 A. M. Waterhouse, J. B. Procter, D. M. Martin, M. Clamp and G. J. Barton, *Bioinformatics*, 2009, **25**, 1189–1191.
